# The use of delta neutrophil index and myeloperoxidase index as diagnostic predictors of strangulated mechanical bowel obstruction in the emergency department

**DOI:** 10.1097/MD.0000000000005481

**Published:** 2016-12-02

**Authors:** Yong Sung Cha, Kang Hyun Lee, Jong Wook Lee, Eun Hee Choi, Hyung Il Kim, Oh Hyun Kim, Kyoung Chul Cha, Hyun Kim, Sung Oh Hwang

**Affiliations:** a Department of Emergency Medicine, Wonju College of Medicine, Yonsei University, Wonju; b Department of Laboratory Medicine, Jincheon Sungmo Hospital, Jincheon; c Biostatistician, Institute of Lifestyle Medicine, Wonju College of Medicine, Yonsei University, Wonju, Republic of Korea.

**Keywords:** delta neutrophil index, mechanical bowel obstruction, myeloperoxidase index, strangulation

## Abstract

Early detection of bowel strangulation is difficult in patients with mechanical bowel obstruction (MBO). There have been no previous reports of predicting strangulation in MBO cases using the delta neutrophil index (DNI), which is a measure of the proportion of circulating immature granulocytes, or the myeloperoxidase index (MPXI), which is a measure of serum myeloperoxidase level. Therefore, we evaluated differences in initial DNI and MPXI upon presentation at the emergency department (ED) according to strangulation presence in MBO patients.

This is a retrospective observational study of consecutive patients older than 18 years who were diagnosed with MBO over a 31-month period. MBO was ultimately confirmed by computed tomography (CT) findings by a radiology specialist. Patients were categorized by a strangulation group (SG) and nonstrangulation group (NSG). The SG was defined by surgical and pathologic findings after the surgical operation. Initial serum counts of white blood cells and neutrophils, C-reactive protein levels, and DNI and MPXI scores were investigated in the ED.

Fifteen of 160 patients were allocated to the SG (9.4%), and among the inflammatory markers, median initial DNI value was the only one that was significantly higher in the SG (0% vs 3.2%, *P* = 0.003). Although the areas under the receiver operation characteristic (ROC) curves for initial DNI and CT for differentiating strangulated from nonstrangulated bowel obstruction were 0.713 (95% confidence interval [CI]: 0.636–0.782) and 0.883 (95% CI: 0.823–0.928), respectively; there was no significant difference between DNI and CT (*P* = 0.147). The area under the curve (AUC) for predicting strangulated bowel disease from a combination of initial DNI score and CT findings (0.983, 95% CI: 0.948–0.997) was higher than the AUC for CT alone, although the difference was not significant (*P* = 0.052).

In conclusion, initial DNI, which was performed in the ED, was found to be significantly higher in the SG than in the NSG. Initial DNI might be a useful additional parameter for improving the prediction accuracy of CT.

## 1 Introduction

Mechanical bowel obstruction (MBO) is a common surgical emergency in the emergency department (ED) and is a major cause of morbidity and financial expense in hospitals around the world.^[1–3]^ A main concern of clinicians treating bowel obstruction is accurate and early recognition of bowel strangulation because strangulation is associated with high morbidity and mortality; however, strangulation detection is very challenging.^[4–6]^

The classical clinical signs of strangulated bowel obstruction include continuous abdominal pain, fever, tachycardia, leukocytosis, peritoneal irritation signs, acidosis, presence of a painful mass, absence of bowel sounds, and blood in the stool.^[6,7]^ Some studies have reported that inflammatory markers such as procalcitonin or intestinal fatty acid-binding protein might be useful for predicting strangulated bowel obstruction.^[8,9]^ Enhanced computed tomography (CT) is a useful tool for differentiating between simple and strangulated bowel obstruction with a high rate of accuracy (73–93%).^[10,11]^ However, the sensitivity and specificity of an intestinal ischemia diagnosis by experienced gastrointestinal radiologists blinded to patient identification and all clinical data range from 15% to 30% and 91% to 94%, respectively.^[12]^ Accordingly, the combined evaluation of clinical, laboratory, and radiologic findings may differentiate simple MBO from strangulated MBO.

The delta neutrophil index (DNI) is a new inflammatory marker that measures the proportion of circulating immature granulocytes.^[13,14]^ Because infectious conditions are associated with increased immature granulocyte levels, several investigators have examined whether this level can be used to predict the development of sepsis.^[15,16]^ On the other hand, the serum myeloperoxidase index (MPXI) is a new index of the level of serum myeloperoxidase (MPO), which is a newly observed inflammatory marker. MPO is released by neutrophils and triggers hypochlorous acid (HOCl) synthesis from hydrogen peroxide and chloride.^[17]^ HOCl plays an important defensive role against bacteria, fungi, and viruses.^[18]^ Furthermore, neutrophils extracted from MPO-deficient individuals show lower microbicidal activity than those extracted from individuals with normal MPO activity.^[18,19]^

MBO can cause bacterial translocation, even in cases of simple obstruction, by promoting bacterial overgrowth, increasing intestinal permeability, and/or physically disrupting the mucosal barrier. Therefore, we thought that DNI and MPXI would be higher in the strangulated group because a strangulated bowel could cause more bacterial translocation, thereby elevating immature granulocyte levels.^[20,21]^ However, no information is available on the clinical usefulness of DNI or MPXI with respect to strangulation diagnosis in patients with MBO.

We evaluated differences in initial DNI and MPXI on presentation at the ED according to presence of strangulation in patients with MBO.

## 2 Materials and methods

### 2.1 Study setting and population

This is a retrospective and observational study of consecutive patients 18 years or older who visited our hospital for MBO over a 31-month period (2012–2014, since DNI analysis was implemented). The ED was located in a single urban tertiary-care hospital that receives more than 43,000 annual visits and is staffed 24 h/d by board-certified emergency physicians.

Any patient whose computerized hospital records included the words “bowel obstruction” or “ileus” in the ED discharge codes was initially considered for study enrollment. Among the selected patients, MBO was suspected based on risk factors (surgical history, hernia, intestinal inflammation, risk of neoplasm), symptoms (vomiting, abdominal pain, constipation), and physical examination findings consistent with obstruction (e.g., abdominal distention). MBO was confirmed with CT findings by a specialized radiologist; confirmed MBO was based on such findings as dilated bowel loops with air–fluid levels, proximal bowel dilation with distal bowel collapse, or a gasless abdomen.^[22,23]^

The study exclusion criteria were hematologic abnormalities or other concurrent infection and those who received granulocyte colony stimulating factors, glucocorticoids, or other immunosuppressants before study enrollment, as these can affect DNI and MPXI; transferred from other hospitals after use of antibiotics because of the possible effects of on DNI and MPXI; transferred to another hospital; discharged against medical advice; had not undergone CT to confirm MBO; and had not undergone emergency life-saving surgery for suspected strangulation.

### 2.2 Data collection

Data were collected by retrospectively reviewing electronic medical records. Data collection was conducted by 2 emergency physicians who were blinded to the study objectives and hypotheses. Abstractors were blinded to the categorization of the patients into either the strangulation group (SG) or nonstrangulation group (NSG), which was performed by another emergency physician. The abstractors were trained to reduce bias during the process prior to data collection. We used explicit case report forms. The chart abstractors and study coordinators met periodically to resolve any disputes and to review coding rules. The study coordinators monitored the performance of the abstractors. Because the study was performed retrospectively and observationally, patient records and/or information were anonymously processed prior to analyses. The following information was obtained from patient medical records: age, sex, time from symptom onset to ED arrival, initial symptoms, initial vital signs, abdominal physical examination, cause and sites of obstruction, CT findings, and presence of strangulation. White blood cell (WBC), neutrophil count, C-reactive protein (CRP), and DNI were measured upon ED arrival.

Patients were categorized into either an SG or an NSG. The SG was defined as patients with surgical and pathologic findings of strangulation. The NSG was defined as patients with pathologic findings that confirmed nonstrangulation or patients who were discharged with conservative management without surgery. Emergency surgery was performed in patients with suspected strangulation, including hemodynamic instability, severe rebound tenderness, identification of bowel ischemia/necrosis by CT, as evidenced by poor or absent segmental bowel wall enhancement, bowel wall thickening, air in the bowel wall (pneumatosis intestinalis), portal or mesenteric venous gas, engorgement of mesenteric vessels, hemorrhage in the mesentery, or ascites.^[11,24]^

The automatic cell analyzer ADVIA 120/2120 (Siemens, Tarrytown, NY) was used to estimate DNI and MPXI values, following the manufacturer's protocol. This is a flow cytometry-based hematologic analyzer that uses 2 independent WBC analysis methods, including an MPO channel and a lobularity/nuclear density channel. We calculated DNI from the leukocyte differentials using the following formula: DNI = (leukocyte subfraction assayed in the MPO channel by cytochemical reaction) − (leukocyte subfraction counted in the nuclear lobularity channel by the reflected light beam).^[25]^ Mean MPXI was determined with the blood autoanalyzer using 4-chloro-1-naphthol (an MPO substrate in granulocytes), and in these cells black precipitates were formed. As stained WBC pass through the flow cell, light scatter (y-axis) and absorbance (x-axis) are measured by a tungsten-halogen light source, and the MPXI is defined by the deviation from the mean neutrophil values on the x-axis.^[26]^ Several biomarkers, such as serum CRP, require additional laboratory processes and expenses, whereas serum DNI and MPXI analyses do not. This is due to the fact that they are routinely performed with leukocyte differential counts in clinical settings, enabling the results to be obtained simultaneously with WBC counts and neutrophil fractions in complete blood count (CBC) testing.

The primary finding in this study was the difference in the initial DNI and MPXI upon presentation to the ED according to the presence of strangulation in patients with MBO. The secondary finding was the ability of initial DNI and MPXI to detect strangulation.

This study was approved by the Institutional Review Board of Wonju College of Medicine, Yonsei University.

### 2.3 Statistical analyses

Categorical variables are presented as frequencies and percentages, and continuous variables are presented as means and standard deviations or as medians and interquartile ranges. Normality was assessed using the Shapiro–Wilk test. We compared the categorical variables with either a chi-squared or Fisher exact test. For continuous variables, we used either 2-sample *t* tests or Mann–Whitney *U* tests. Each method's ability to detect strangulation presence was evaluated using the area under the receiver operating characteristic (ROC) curve. Additionally, the ability of each method to predict the presence of strangulated bowel obstruction was compared using the area under the curve (AUC). *P*-values <0.05 were considered statistically significant, and all analyses were performed using SPSS version 23 (IBM, Armonk, NY) or SAS 9.2 (SAS Institute Inc, Cary, NC).

## 3 Results

### 3.1 Characteristics of study subjects

A total of 212 consecutive MBO patients, all older than 18 years, were identified during the study period. Patients were excluded for the following reasons: another concurrent infection, including pneumonia, urinary tract infection, or intraperitoneal abscess (5 patients); had been transferred from another hospital after use of antibiotics (15 patients); had to transfer to another hospital (5 patients); were discharged against medical advice (2 patients); did not undergo CT scan (15 patients); did not undergo emergency surgery (2 patient); or had insufficient data (8 patients). Ultimately, our study included 160 of a total of 212 MBO patients.

The baseline characteristics of the 160 study subjects are shown in Table 1. Ninety-six patients were male (60%), and the overall median age was 69 years. The median time from symptom onset to ED arrival was 24 h. Common initial symptoms at ED presentation were abdominal pain (98.8%), no passage of gas (82.2%), and vomiting (62.6%). Abnormal abdominal physical examination findings included abdominal tenderness (71.2%), abdominal distention (50.4%), and rebound tenderness (11.9%). Seventeen patients (10.6%) were suspected to have strangulation of the bowel based on CT and emergency surgery was performed in 23 patients (14.4%). The most common cause of strangulation was adhesion (66.9%), followed by carcinoma (16.3%), and bowel inflammation (9.4%). One hundred twenty-two patients (77.7%) had small bowel obstruction (Tables 1 and 2).

**Table 1:**
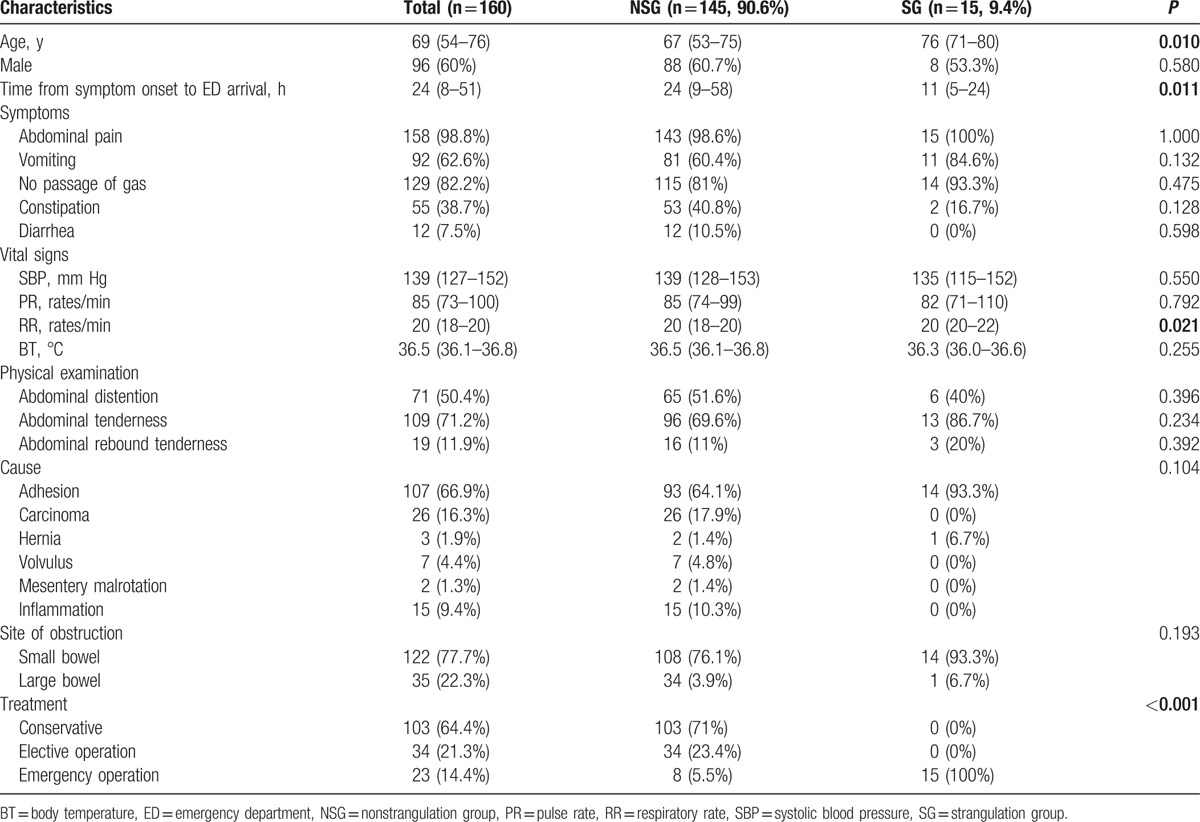
Baseline characteristics of patients with mechanical bowel obstruction.

**Table 2:**
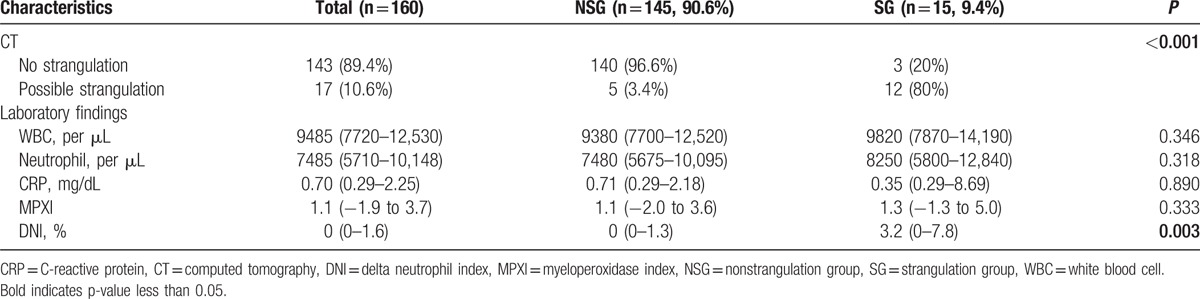
Laboratory and computed tomography findings from patients with mechanical bowel obstruction.

The SG included 15 patients (9.4% of the entire group and 65.2% of patients who underwent emergency surgery). Twelve (80%) of these 15 patients with strangulation were diagnosed by CT findings (*P* < 0.001). The results of the univariate analysis comparing the NSG and SG are shown in Table 1. Patients in the NSG and SG differed significantly in terms of age (67 years vs 76 years, *P* = 0.010), time from symptom onset to ED arrival (24 h vs 11 h, *P* = 0.011), and respiratory rate (20 rates/min vs 20 rates/min, *P* = 0.021) (Tables 1 and 2).

### 3.2 Main results

**Figure 1:**
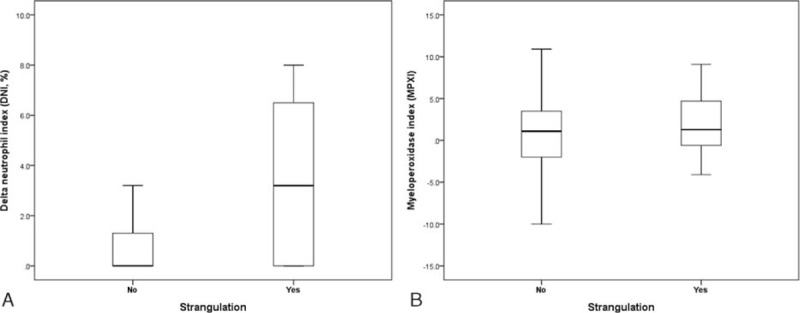
Median values of delta neutrophil index (A) and myeloperoxidase index (B) according to strangulation presence.

**Figure 2:**
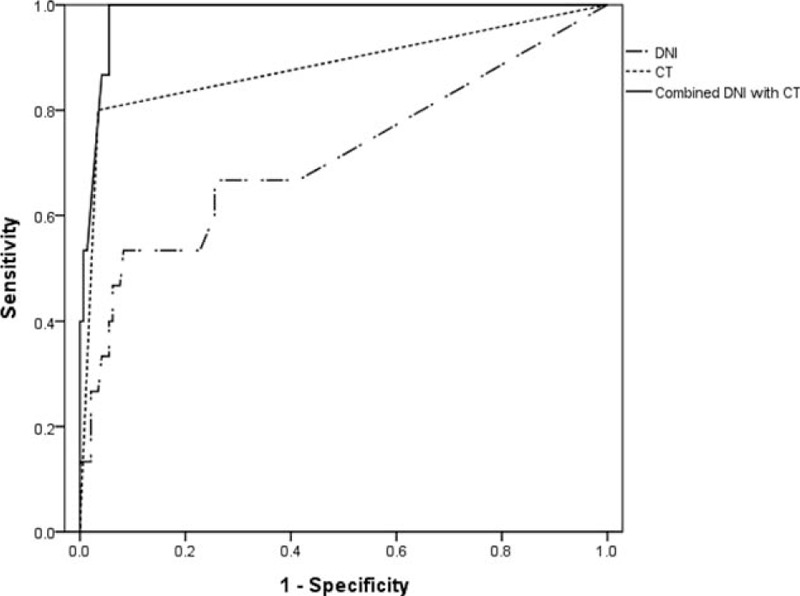
Receiver operator characteristics of delta neutrophil index, computed tomography, and combined delta neutrophil index with computed tomography. CT = computed tomography, DNI = delta neutrophil index.

The median initial DNI value was the only factor that was significantly higher in the SG compared with the NSG (0% vs 3.2%, *P* = 0.003) (Table 2; Fig. 1). Although the areas under the ROC curve for initial DNI and CT for differentiating strangulated from nonstrangulated bowel obstruction were 0.713 (95% confidence interval [CI]: 0.636–0.782) and 0.883 (95% CI: 0.823–0.928), respectively, there was no significant difference between DNI and CT (*P* = 0.147). The AUC for predicting strangulated bowel disease using a combination of initial DNI and CT (0.983 [95% CI: 0.948–0.997]) was higher than the AUC for CT alone, although there was no significant difference (*P* = 0.052; Table 3; Fig. 2).

**Table 3:**
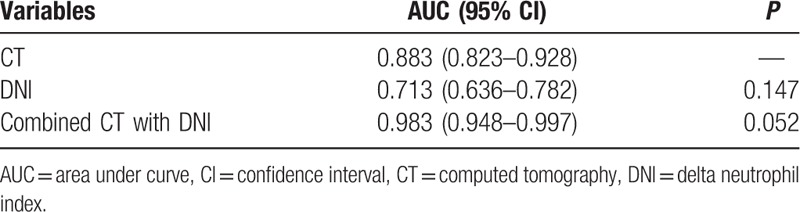
Areas under the curve for prediction of strangulated bowel obstruction according to delta neutrophil index and computed tomography and comparisons of areas under the curve for combined variables.

In this study, of 15 patients in the SG, 3 (20%) had strangulated bowel, as confirmed during surgery, but had no findings of strangulation on CT (i.e., false-negative CT finding). The median initial DNI of these patients was 8%. Of the 145 patients in the NSG, 5 also had confirmed nonstrangulated bowel but had findings of strangulation on CT (i.e., false-positive CT finding). The median initial DNI of these patients was 0%.

## 4 Discussion

In this study, initial DNI on presentation to the ED was significantly greater in patients with strangulation compared to those without strangulation. Also, the prediction ability of initial DNI for strangulated bowel obstruction was not statistically different from the prediction ability of CT (*P* = 0.147). MBO can cause bacterial translocation, even in cases of simple obstruction, by promoting bacterial overgrowth, increasing intestinal permeability, and/or physically disrupting the mucosal barrier. Therefore, immature granulocytes may be elevated in the MBO, regardless of strangulation. Also, in cases of MBO with strangulation, immature granulocytes may be elevated because a strangulated bowel could cause increased bacterial translocation.^[20,21]^ Nahm et al^[25]^ showed that DNI was strongly correlated with manual immature granulocyte count (*r* = 0.75, *P* < 0.05). Therefore, DNI was higher in the SG, indicating its diagnostic value.

We also found that the median initial DNI of patients with false-negative CT findings was high (8%), while the median initial DNI was low (0%) in patients with false-positive CT findings. Because it may be difficult to differentiate simple bowel obstruction from strangulated bowel obstruction with a single diagnostic method, we evaluated the predictive ability of abdominal CT combined with initial DNI. CT combined with initial DNI (AUC = 0.983) predicted strangulated bowel obstruction better than CT alone (AUC = 0.883) and had the highest diagnostic power in our study (Table 3). DNI analysis does not require any additional time or cost in the clinical setting because it is performed routinely with leukocyte differential counts, and the results can be obtained at the same time as WBC counts and neutrophil fractions from CBC testing. Even though initial DNI cannot by itself replace careful clinical examination and CT, we suggest that it could be a useful addition to improve the predictive accuracy of CT.

That said, compared to NSG, MPXI was not significantly higher in the SG. Yonezawa et al^[26]^ found that elevated MPXI indicates increased MPO activities microbicidal activities in an infectious state. However, in severe bacterial infections such as sepsis, the activated neutrophils release large amounts of MPO for bactericidal activities, and an increase in degranulated neutrophils can reduce the MPXI values. This is because the MPXI level is likely controlled by the synthesizing and release of MPO. Therefore, we concluded that under severe septic conditions due to bowel strangulation, MPXI might not undergo a significant increase.

Serum CRP is widely used as an objective index of disease activity in ED settings. The concentration of CRP, a plasma protein, rises dramatically as a result of cytokine-mediated responses to most forms of infection, inflammation, and tissue injury.^[27]^ In this study, serum CRP was not significantly different between the NSG and SG, as was found in the study of Kittaka et al.^[9]^ Also, Simon et al reported that, for bacterial infections, serum CRP has little sensitivity and specificity.^[28]^ We feel that further study is needed to determine the usefulness of CRP in this regard.

Median age differed between the patient groups in this study. Patients with strangulation tended to be older, which may be because older patients are more likely to have decreased physiologic defense mechanisms to bowel ischemia and necrosis. In this study, the SG had a shorter time between symptom onset and ED arrival than the NSG. The SG demonstrated more severe symptoms that occurred more rapidly and more severely compared to the NSG; this heightened severity may have caused them to come to the ED sooner than patients in the NSG. However, other reports found no difference in terms of time of symptom onset to ED arrival between SG and NSG.^[8,9]^

This study has some limitations. First, it has a retrospective design. Many types of data are missing, especially those regarding initial symptoms and data from physical examination (e.g., rebound tenderness), and laboratory tests (e.g., initial serum lactate or base excess). Several experimental studies have demonstrated that lactate level is a useful predictor of intestinal ischemia, and clinical reports have described this parameter as a significant biomedical marker for diagnosing strangulated small bowel obstruction.^[29,30]^ In this study, although initial serum lactate level was significantly higher in patients in the SG than in those in the NSG (2.46 vs 1.09, *P* = 0.001), initial serum lactate was only measured in 93 of 160 patients (58.1%). Therefore, we do not present results for initial serum lactate level because it was difficult to compare DNI or MPXI with lactate level. Second, since this study was conducted at the emergency center of a single hospital, the sample size was small. Nevertheless, we investigated all MBO patients who were admitted to this hospital since DNI and MPXI levels became measureable in order to reduce possible biases. Third, DNI and MPXI values can be affected by infection or inflammation out of cause of MBO as well as presence of strangulation. However, there were no differences in the median results for DNI or MPXI values between the NSG and SG when we analyzed inflammatory markers after controlling for inflammation (DNI: 0% vs 3.2%, *P* = 0.001 and MPXI: 1.1 vs 1.3, *P* = 0.252). Fourth, because serial DNI and MPXI values were not investigated postoperatively, we did not evaluate their usefulness for evaluating treatment responses by assessing changes in inflammatory markers after definitive treatment. Therefore, a prospective study of DNI is needed to expand our current ability to predict complications and serial results after surgery.

In conclusion, initial DNI, which was measured in the ED, was found to be significantly higher in the SG than in the NSG. Initial DNI might be a useful additional parameter that could improve the prediction accuracy of CT.
